# Supplementary figures and images for: DengueSeq: a pan-serotype whole genome amplicon sequencing protocol for dengue virus
Source: BMC Genomics. 2024 May 1;25:433. doi: 10.1186/s12864-024-10350-x (PMC11062901; doi:10.1186/s12864-024-10350-x)

# Inputs

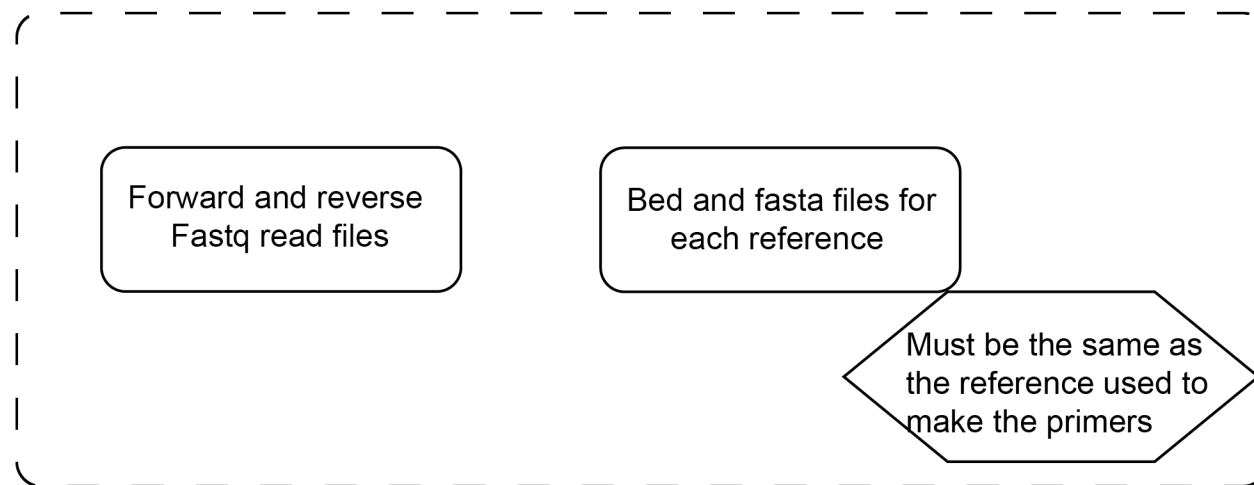

## Consensus generation

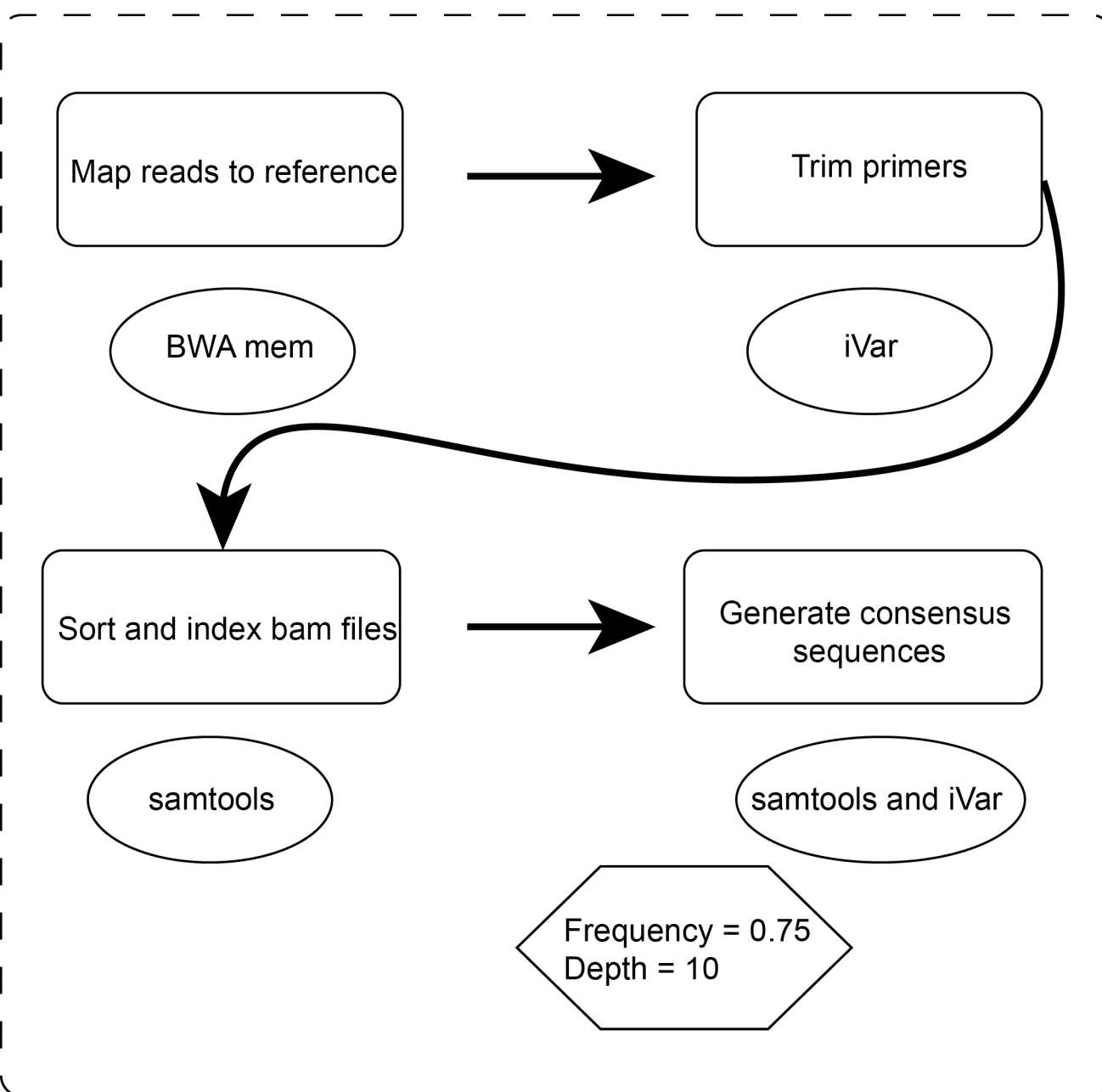

## Get virus calls

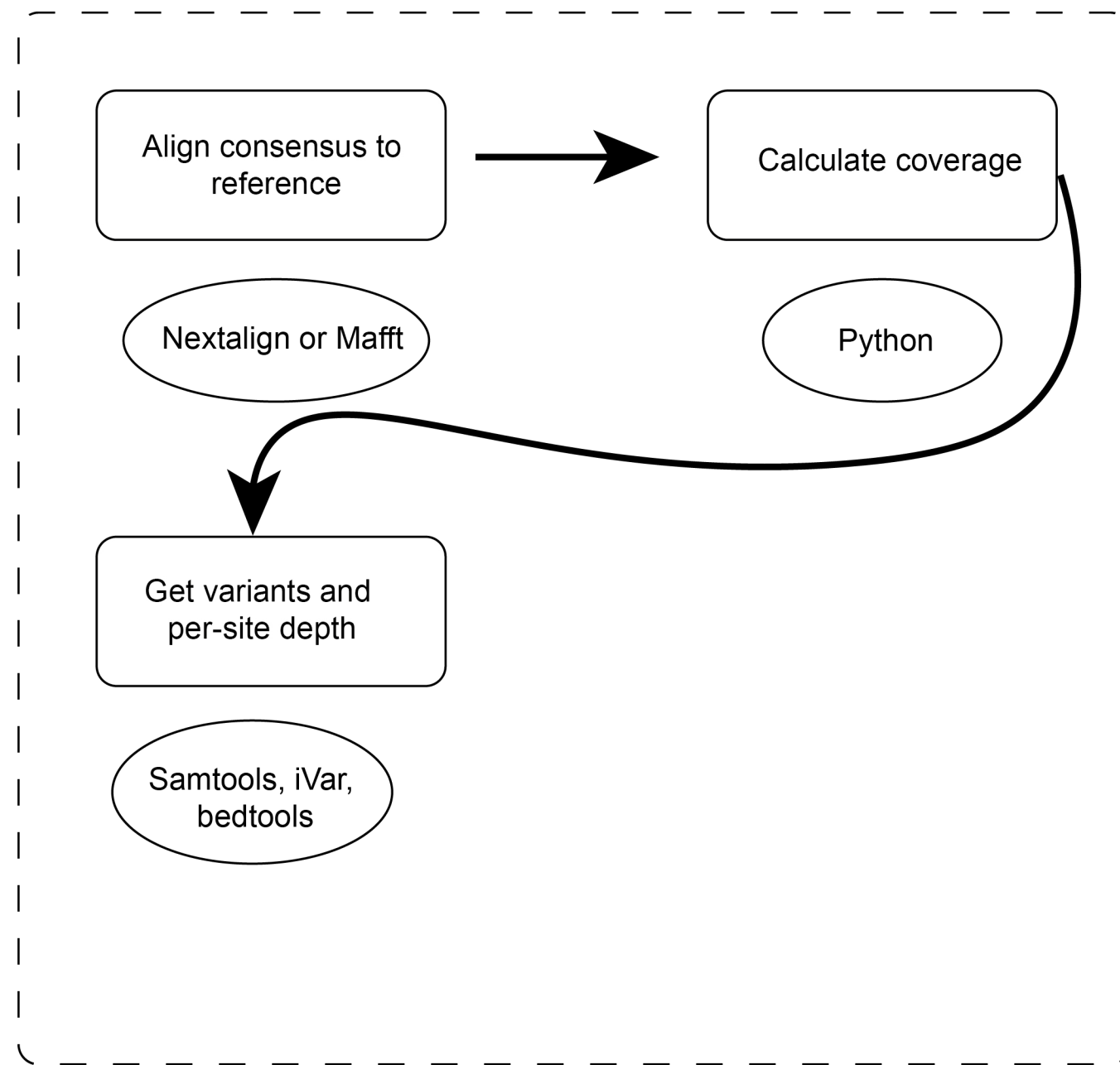

# Outputs

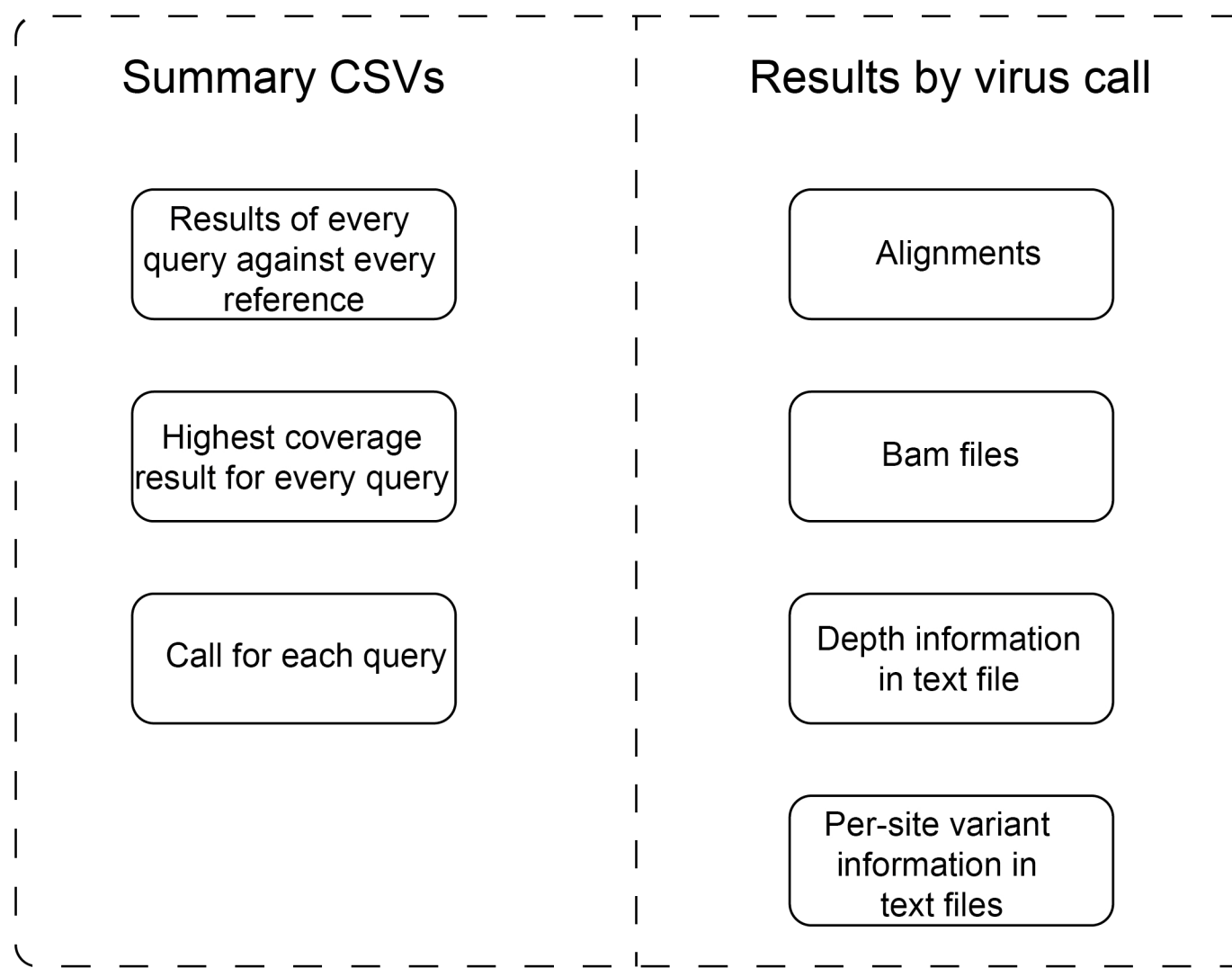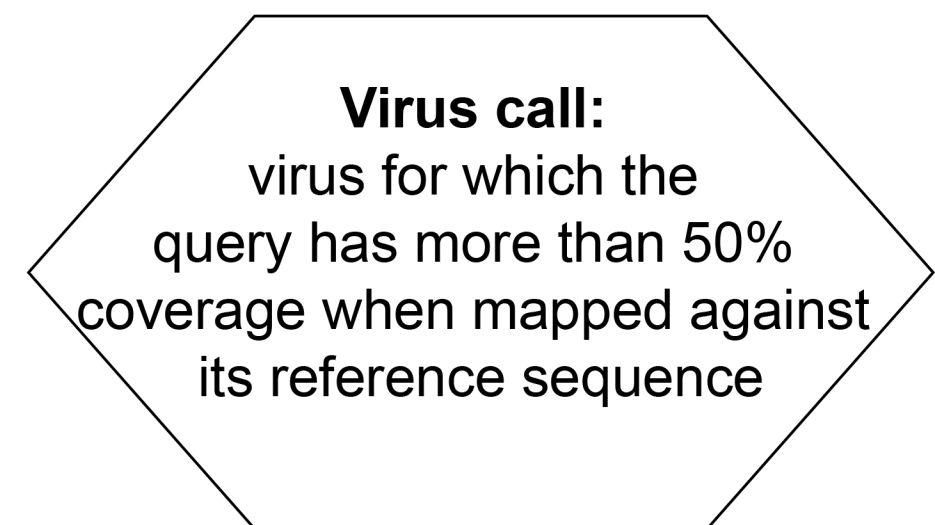

Supplement: Supplementary file 2 — Supplementary Material 2. [file 12864_2024_10350_MOESM2_ESM.pdf]

DENV1

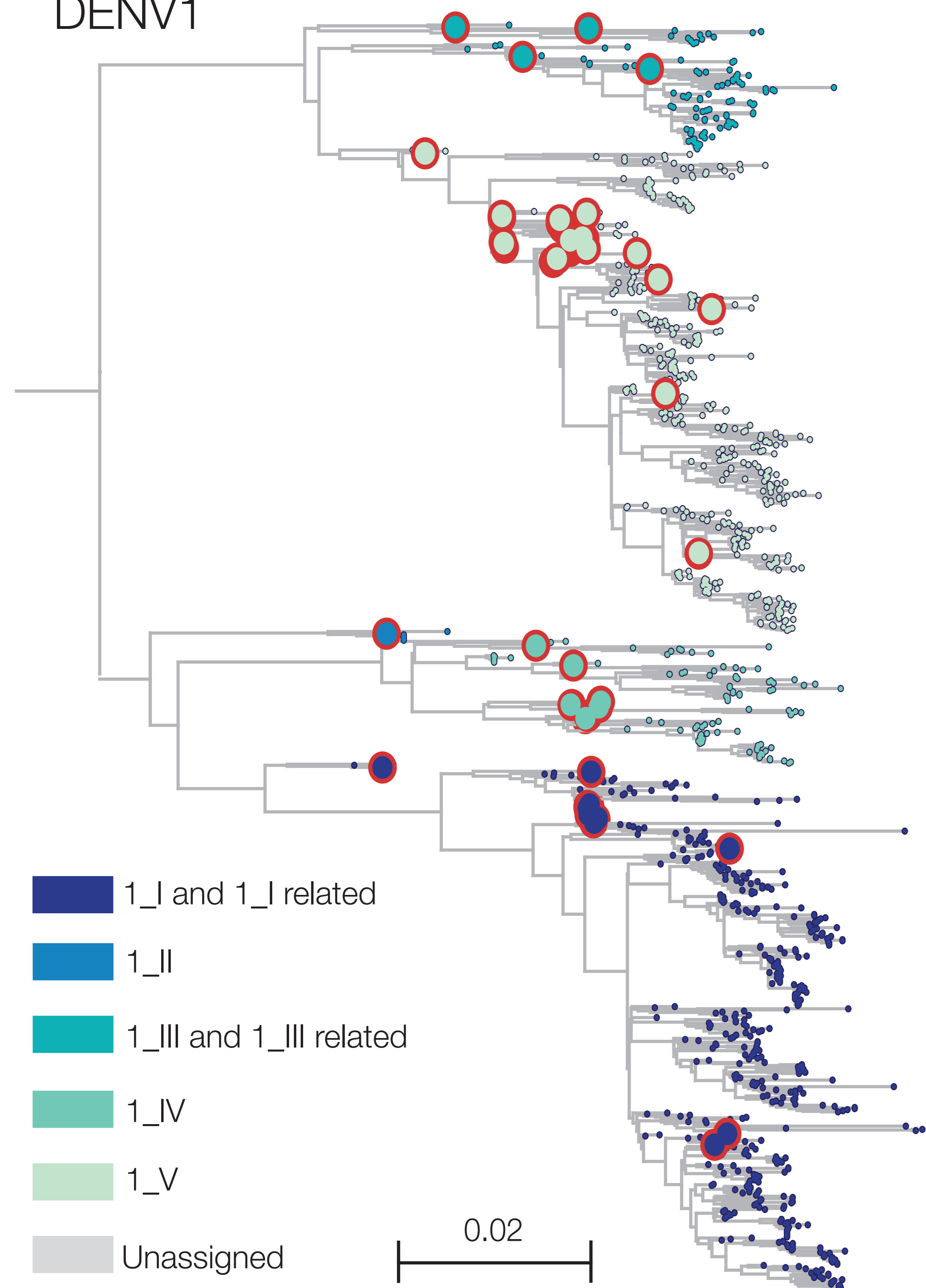

DENV2

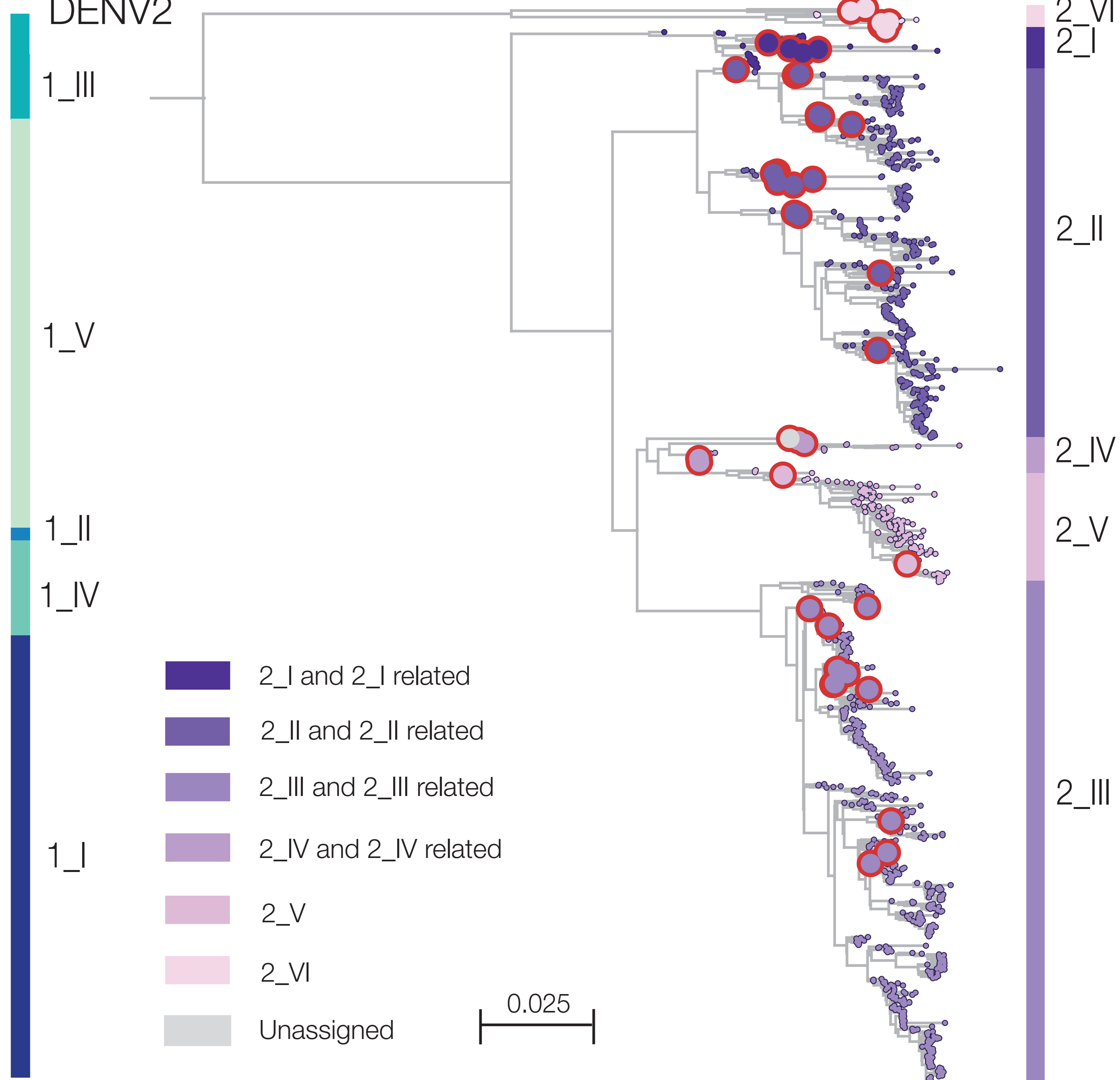

DENV3

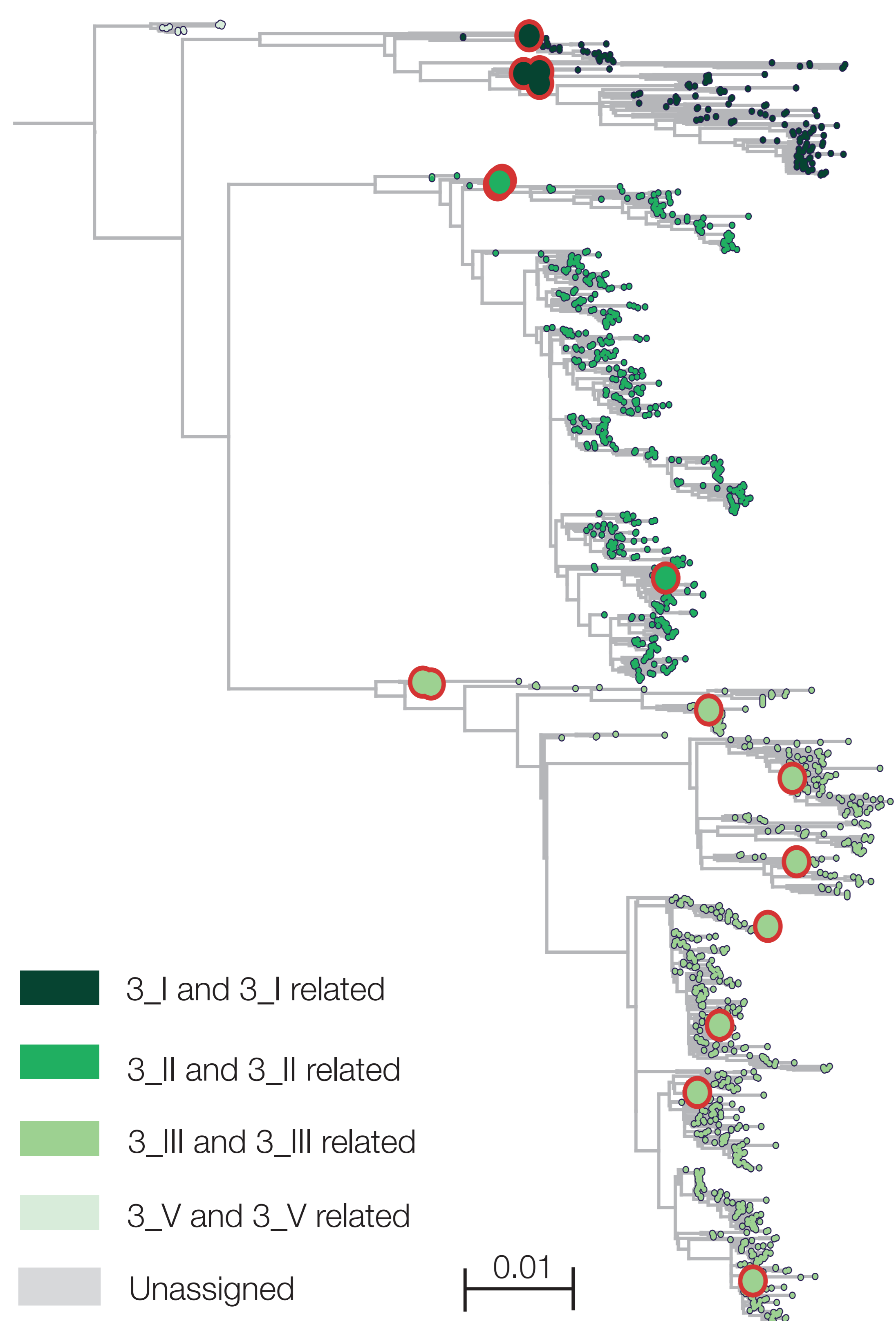

DENV4

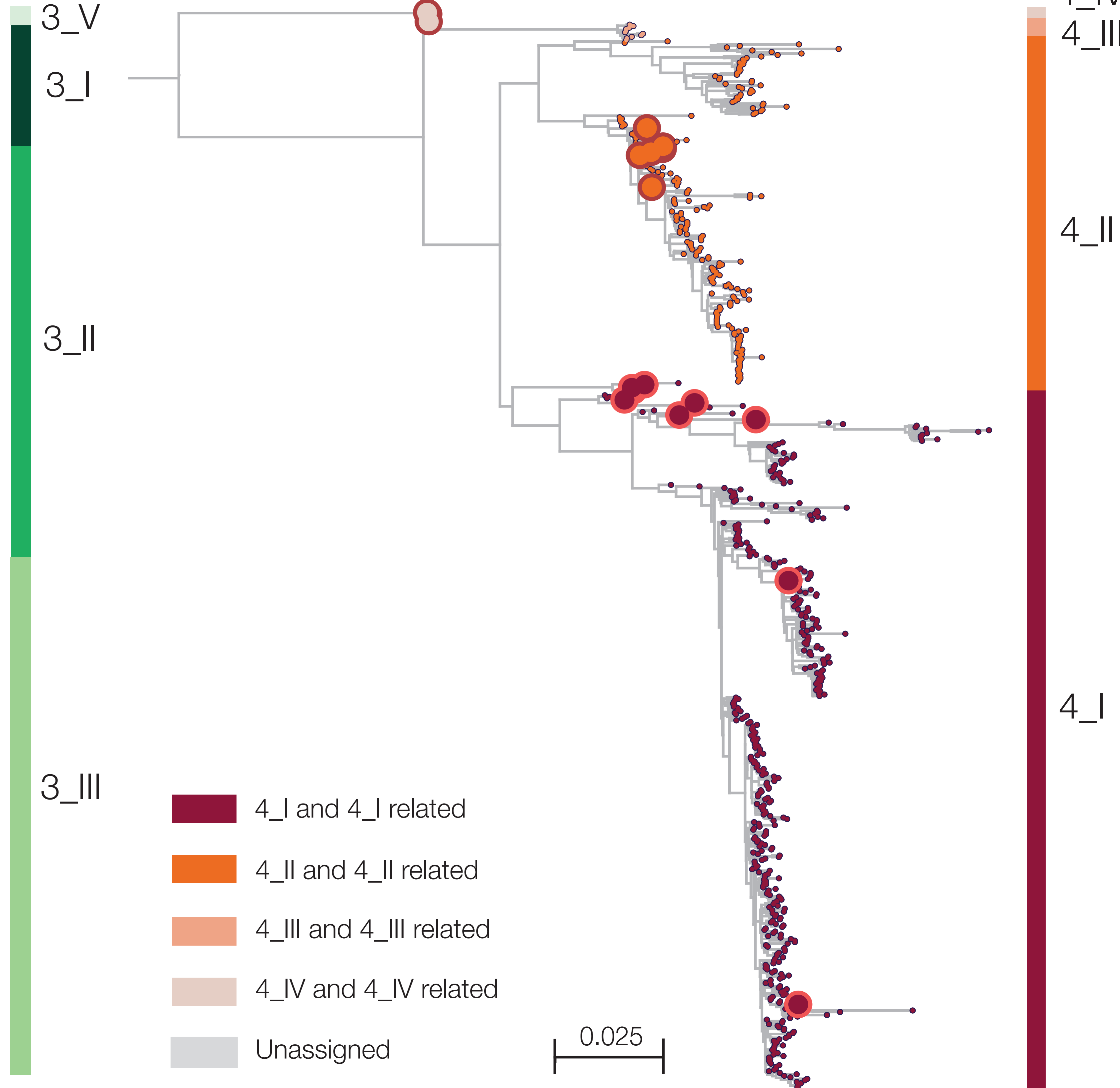

Supplement: Supplementary file 4 — Supplementary Material 4. [file 12864_2024_10350_MOESM4_ESM.pdf]

DENV1

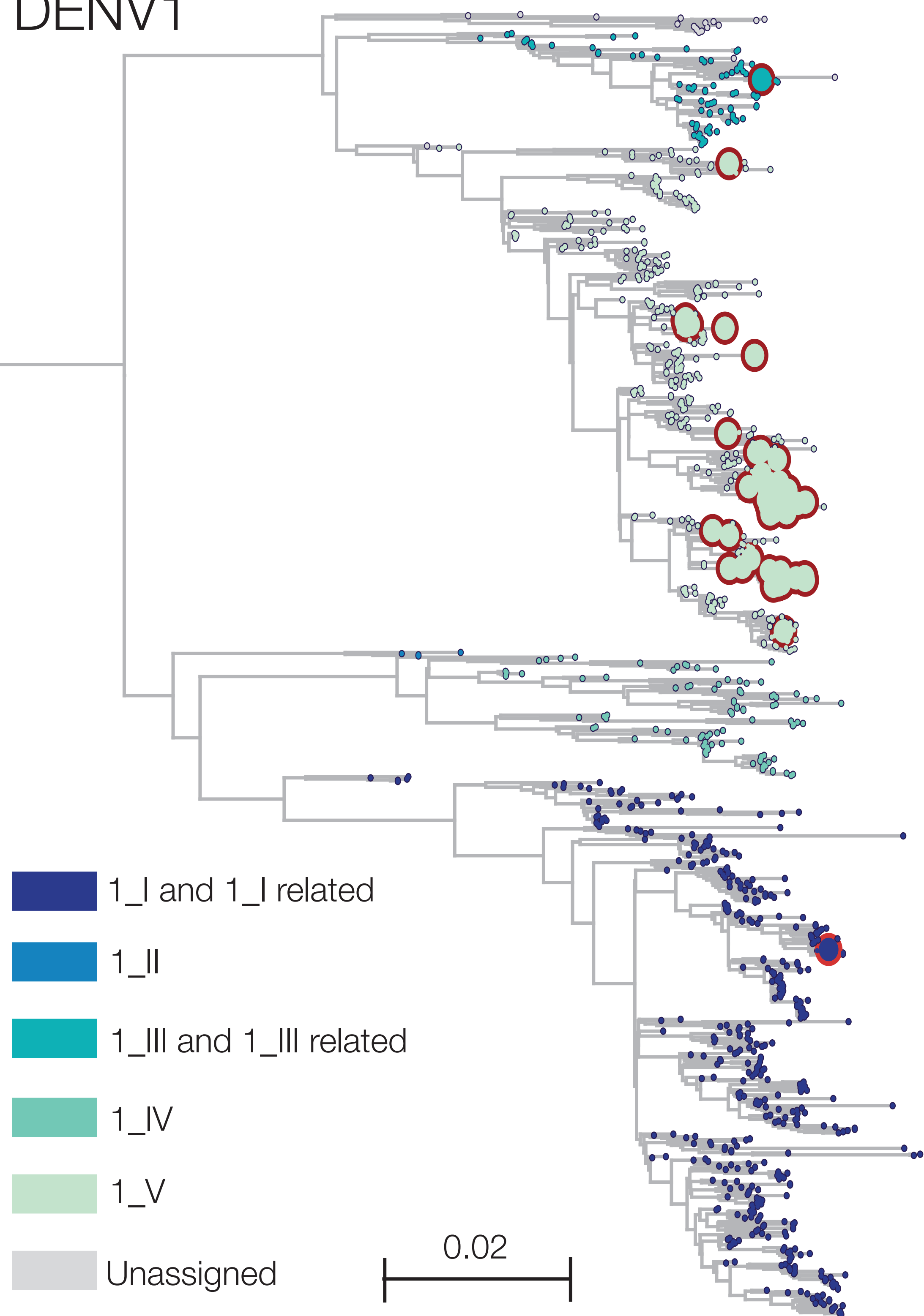

DENV2

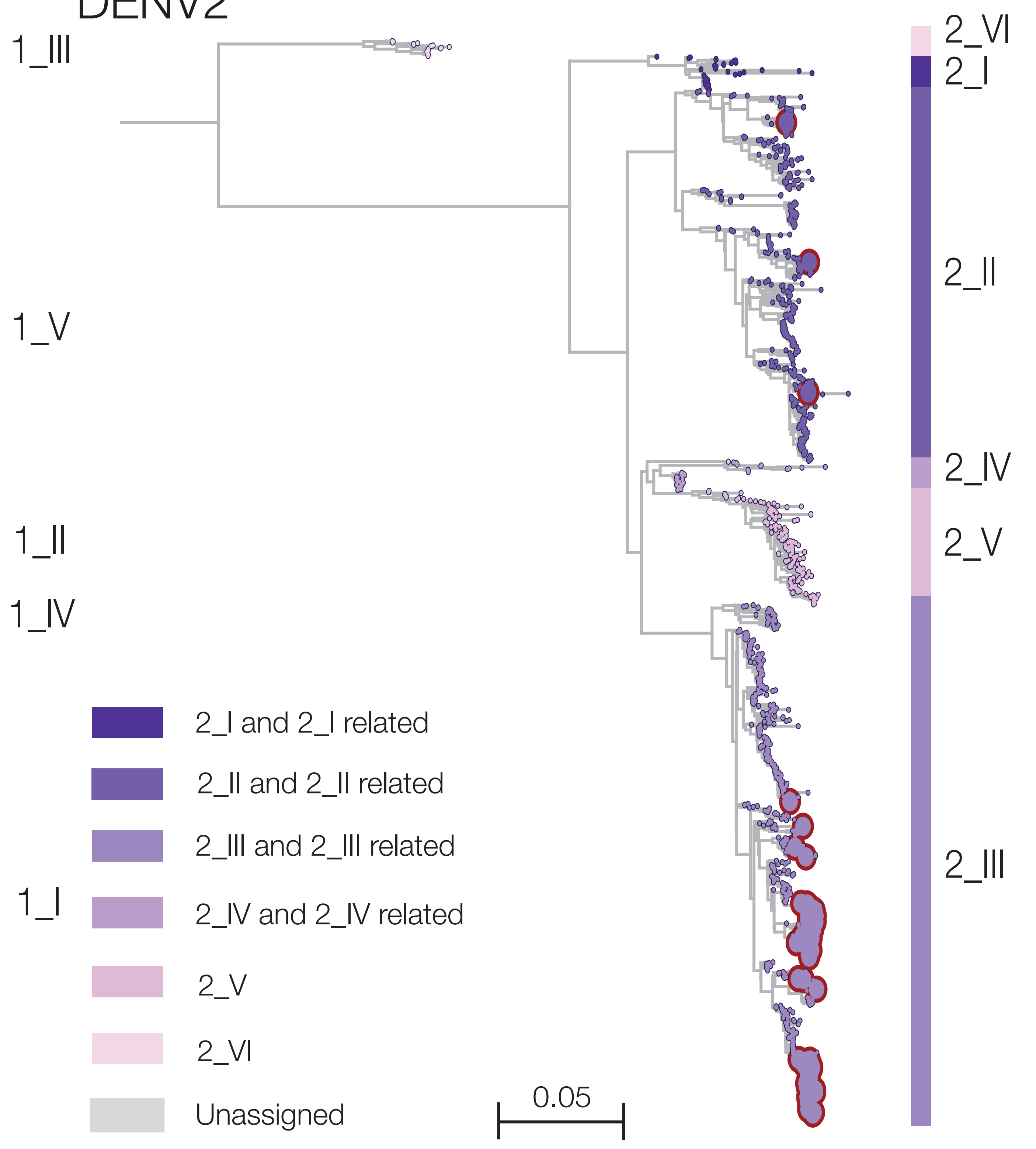

DENV3

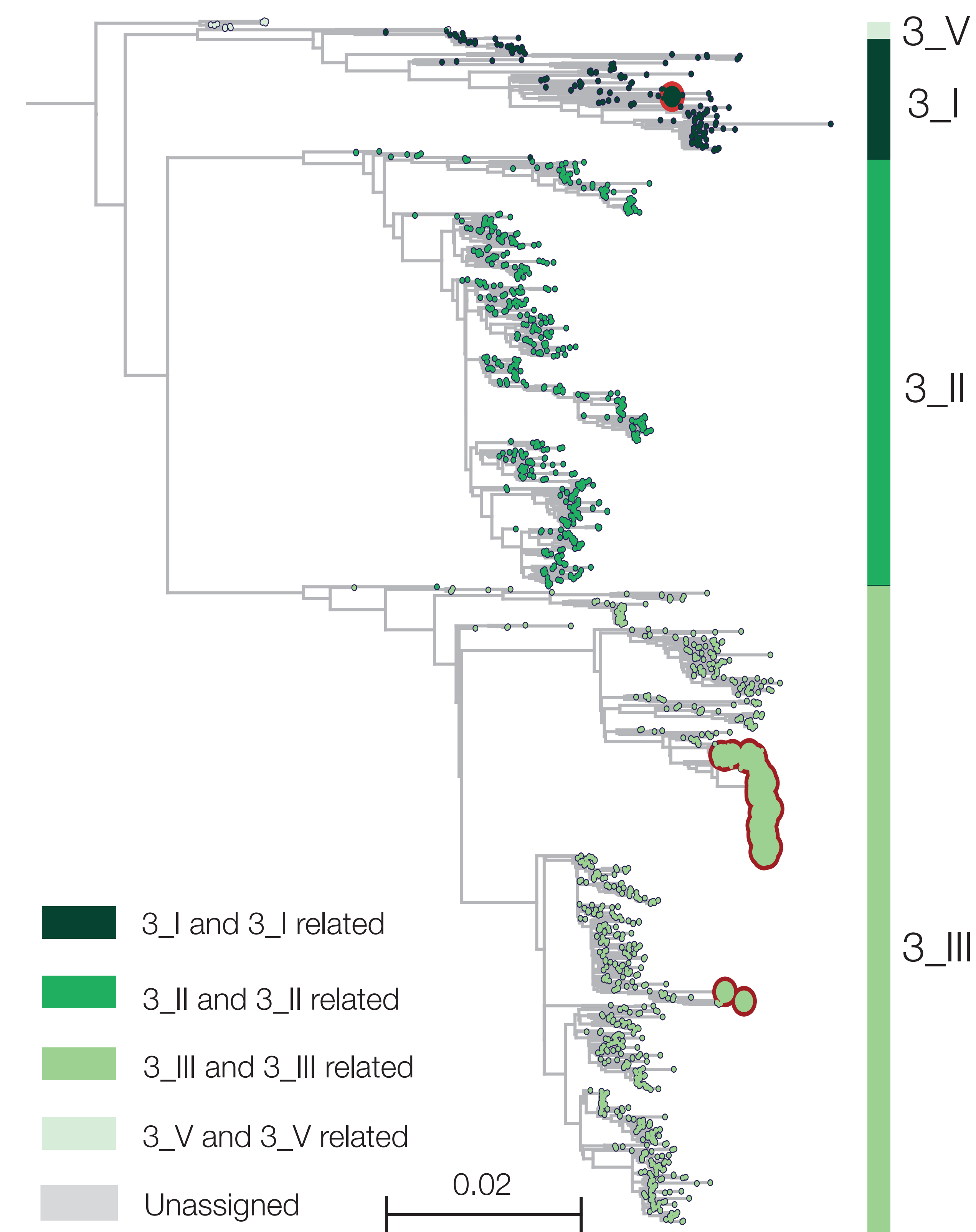

DENV4

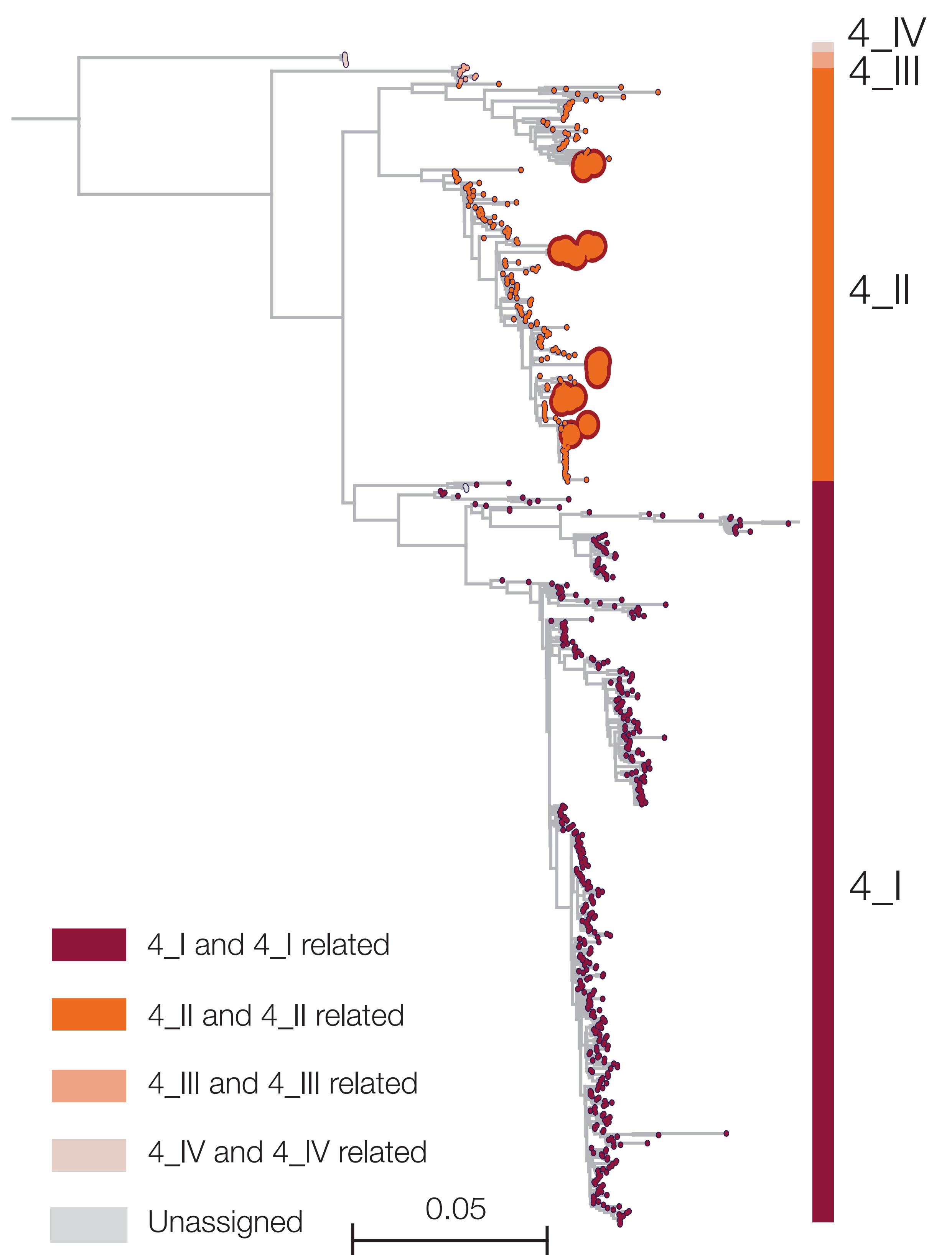

Supplement: Supplementary file 5 — Supplementary Material 5. [file 12864_2024_10350_MOESM5_ESM.pdf]

### Genome coverage at 20X (%)

# Virus stocks

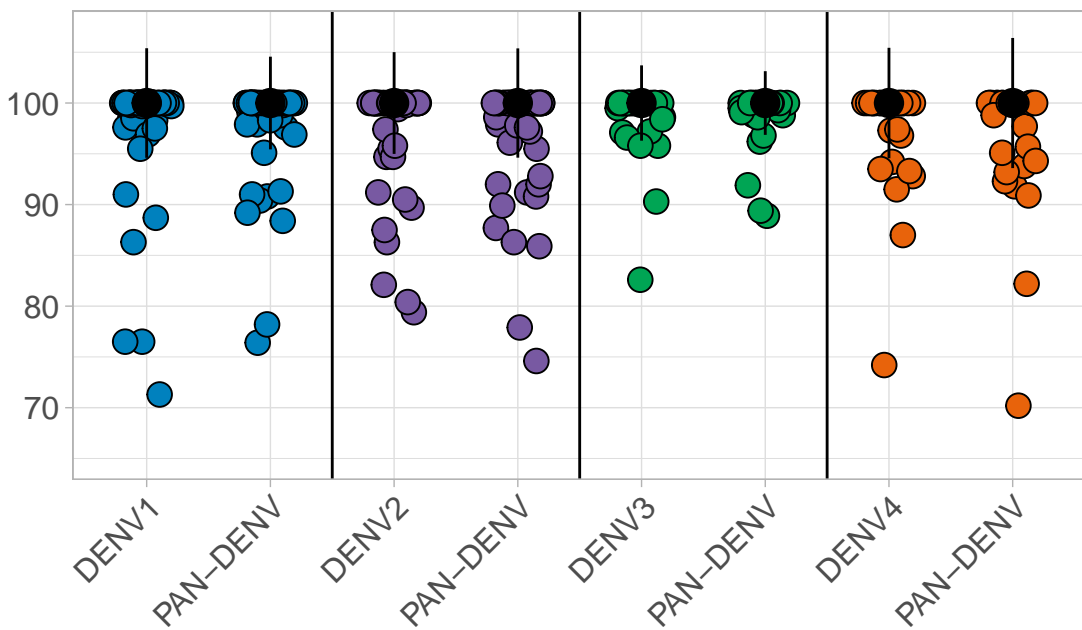

**Genome coverage at 20X (%)**

## Clinical Samples

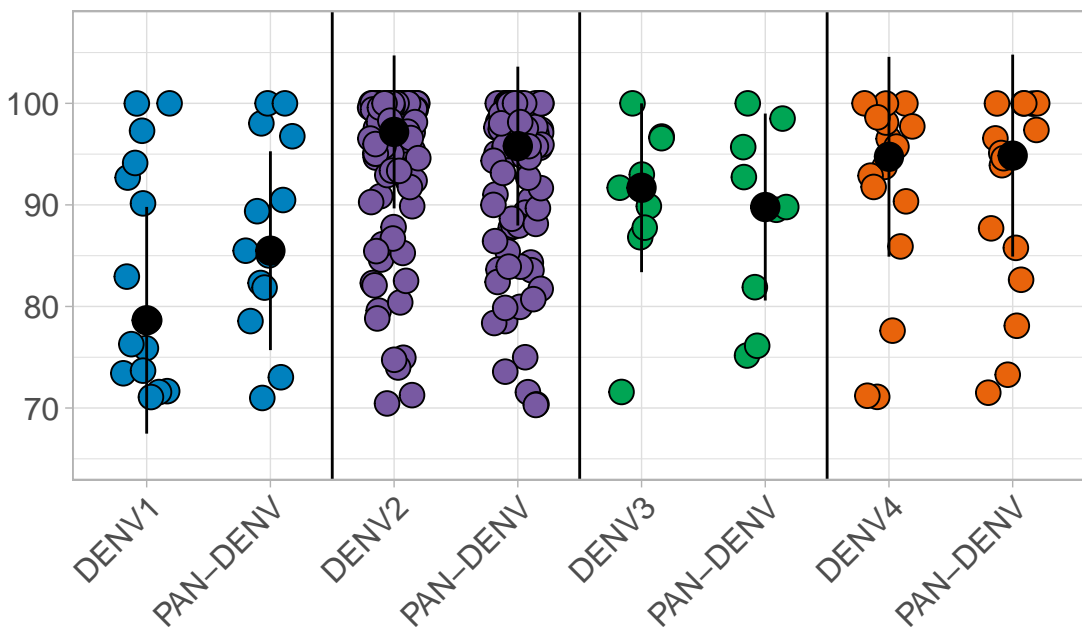

Supplement: Supplementary file 6 — Supplementary Material 6. [file 12864_2024_10350_MOESM6_ESM.pdf]
