# Supplementary material for: DengueSeq: a pan-serotype whole genome amplicon sequencing protocol for dengue virus
Source: BMC Genomics. 2024 May 1;25:433. doi: 10.1186/s12864-024-10350-x (PMC11062901; doi:10.1186/s12864-024-10350-x)
Supplement: Supplementary file 3 — Supplementary Material 3. [file 12864_2024_10350_MOESM3_ESM.pdf]

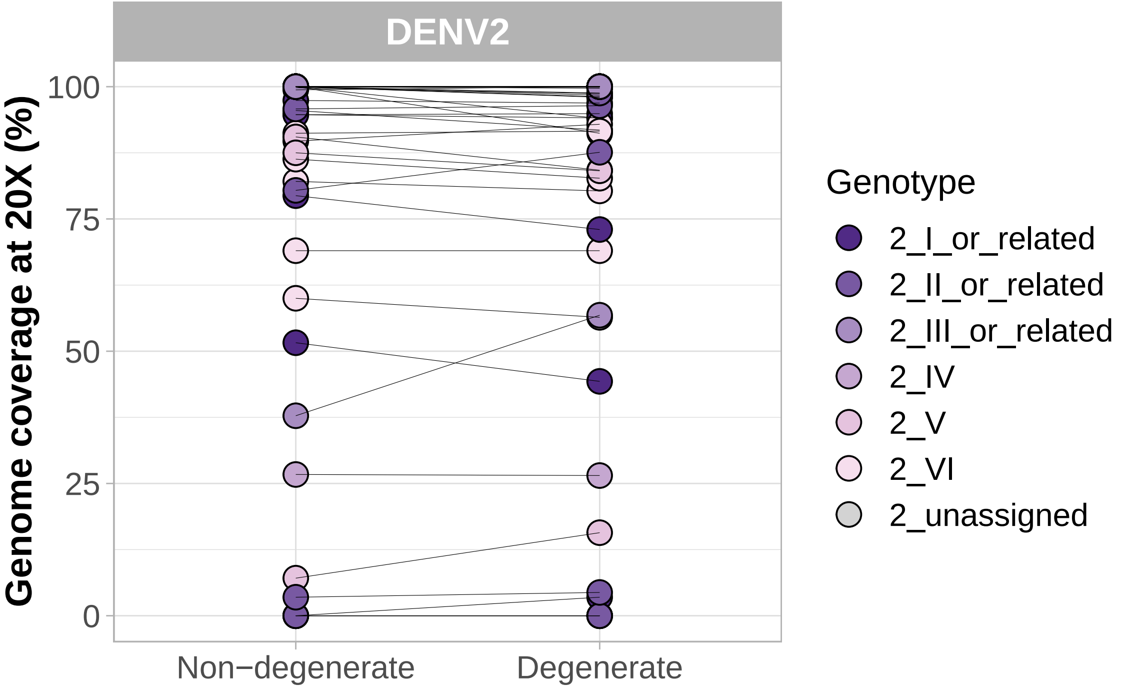
DENV2
Genome coverage at 20X (%)
100
75
50
25
0
Non−degenerate
Degenerate
Genotype
2_I_or_related
2_II_or_related
2_III_or_related
2_IV
2_V
2_VI
2_unassigned
